# Iron status in piglets at three days of age and at weaning and possible seasonal effects on the blood haemoglobin levels in a Swedish outdoor pig-producing farm

**DOI:** 10.1186/s13028-024-00735-z

**Published:** 2024-03-19

**Authors:** Johanna Fjelkner, Axel Sannö, Ulf Emanuelson

**Affiliations:** 1 Farm & Animal Health, SE-245 22 Staffanstorp, POB 164, Sweden; 2 Farm & Animal Health, Kungsängens Gård, SE-753 23 Uppsala, Sweden; 3 Department of Clinical Science, Swedish University of Agricultural Sciences, SE- 75007 Uppsala, POB 7054, Sweden

**Keywords:** Iron deficiency anaemia, Suckling piglet, Swine

## Abstract

**Background:**

Piglets are born with limited stores of iron, and with an increasing number of live-born piglets, there may be a risk that the sows cannot provide enough iron to their offspring. The iron content in soil may not meet the demands of today’s piglet, born and reared in an outdoor setting. The study aimed to describe the blood haemoglobin (Hb) levels in pigs reared outdoors and to determine whether piglets have higher Hb levels at weaning when an iron supplement is administered intramuscularly at three days of age, as compared to pigs not given an iron supplement. The seasonal variation in Hb-levels was also to be investigated. The Hb concentration was analysed with a HemoCue 201 + Hb photometer.

**Results:**

In total 56 litters (399 piglets) were included in the study and sampled at three days of age, while 378 piglets were sampled at weaning. The mean Hb level at three days of age was 91 g/L (48–154 g/L). In total 47% of the piglets had Hb levels < 90 g/L at three days of age. The mean Hb level at weaning was 127 g/L (76–176 g/L), with a lower level (122 g/L) in the group given the iron supplement than in the group not given an iron supplement (132 g/L). Only 1% of the piglets had Hb levels lower than 90 g/L at weaning. Results indicative of a seasonal effect on Hb levels at three days of age was demonstrated. Piglets born in spring had significantly lower Hb levels, and piglets born in autumn had significantly higher Hb levels. No seasonal effect could be demonstrated for Hb levels at day 33.

**Conclusions:**

The results indicate that the natural uptake from the environment was sufficient, but that there was a seasonal effect on the Hb levels at three days of age. This indicates that there might be a need for different routines regarding iron supplementation in outdoor reared piglets depending on the climate and season.

## Background

Iron is a critical constituent of haemoglobin (Hb) for oxygen transport by the blood, and it is also a part of myoglobin and important for various enzymes in the body [1]. The fast increase in piglet body weight during the first weeks of life is accompanied by an expansion of blood volume and a high erythropoietic activity, resulting in an increased number of red blood cells that require a large amount of iron to maintain adequate Hb levels [2, 3]. Piglets are born with limited levels of iron stored in the liver (40 to 50 mg Fe) [4] and the levels of iron may vary between different piglets in the same litter [1]. After birth, the piglet iron requirement is estimated to be approximately 7 to 10 mg of iron per day [3]. However, the amount of iron provided from the sow’s milk ranges from 1.27 to 4.6 g/L which is insufficient [5] and unless the piglet acquires iron by another route, iron deficiency anaemia (IDA) will occur [2, 3].

Piglets born indoors are therefore commonly given a supplement of iron during their first week of life [1, 3, 6]. Most authors agree that piglets show signs of anaemia at Hb levels of 80 g/L or lower [1]. In a study investigating early indicators of iron deficiency in piglets at weaning, the Hb levels were categorized as low (Hb < 90 g/L), medium (90 to 110 g/L) or high (Hb > 110 g/L) [7]. This categorization of Hb levels might be of use in veterinary practice to identify piglets at risk of developing IDA [8].

On the other hand, it is suggested that outdoor-reared piglets do not need iron supplementation since they have access to naturally occurring iron in the soil [4, 9–12] and may also obtain iron from plants and bedding material [10]. Iron is a relatively abundant element of the soil with a total concentration of 20–40 g/kg [13]. Studies have shown that the blood haemoglobin levels at weaning in piglets born outdoors were similar to, or even exceeded, the values in piglets born indoors and receiving an iron supplementation (weaning at 28 and 35 days of age respectively) [10, 11]. On the other hand, there are also studies showing significantly higher blood haemoglobin levels at weaning (approximately 26 days of age) in piglets born outdoors given iron supplementation at 48 h of age, as compared to piglets born and reared outdoors without being provided any iron supplementation [14, 15]. The rapidly increasing number of live-born piglets may further increase the risk that the sows cannot provide enough iron to their offspring [3]. The Swedish national average of live-born piglets in year 2000 was 11.3, as compared to 15.0 in 2020 [16]. It is therefore not clear if the natural supplement of iron from the soil is sufficient to meet the demands of the piglet of today, born and reared in an outdoor setting. In addition, there might be a seasonal variation in blood haemoglobin levels in piglets born outdoors. During winter, the ground is often frozen, and the sow may spend more time inside the farrowing huts [15].

The overall aim of this study was to describe the blood haemoglobin levels in piglets at three days of age and at weaning in a Swedish outdoor pig-producing farm, and the effect of different management strategies regarding the prevention of IDA. The levels of blood haemoglobin at weaning when an iron supplement was administered at three days of age, was compared to the levels in pigs not given an iron supplement and the seasonal variation in blood haemoglobin levels were investigated.

## Methods

### Housing and management

The study was conducted between June 2020 and June 2021 in a Swedish, outdoor, pig-producing herd situated in the southern part of Sweden. The average temperature in this area during winter (i.e. December through February) was − 3 °C, and during summer (i.e. June through August); +18 °C [17].

The herd kept 200 sows divided in seven batches, farrowing at three-week intervals in a batch-wise production system. The piglets were crosses of TN70-sows (TopigsNorsvin; Helvoirt, The Netherlands) inseminated with DanBred Duroc (DanBred P/S; Ballerup, Denmark). Breeding of both gilts and sows was done by artificial insemination using fresh semen purchased from a Swedish boar stud. The sows were routinely vaccinated against *Escherichia coli*, Porcine parvovirus, *Erysipelothrix rhusiopathiae* and Influenza A virus 2009 (H1N1) pdm09. The piglets were routinely vaccinated against porcine circovirus 2 (PCV2), *Mycoplasma hyopneumoniae* and *Lawsonia intracellularis* at weaning. Sweden is declared free from PRRS and thus, no measures to combat this disease were undertaken. The standard routine on the farm was to not provide any iron supplementation to the piglets.

During farrowing, the sows were housed outdoors with access to farrowing huts, allocated in six paddocks (3000 to 6000 m^2^ each) separated by electric fences. Fenders were placed in the front of each hut until one week after farrowing. The farmer provided the sows with a straw bed in the hut and added fresh straw on top of the existing bedding every day during the lactation period. The sows and piglets had *ad libitum* access to water and feed. The piglets were fed a commercial creep feed (150 mg iron/kg feed) from two weeks of age and had access to the sow-feed (80 mg iron/kg feed) during the entire suckling period.

Cross-fostering was practised to equalise the litter size at birth. Surgical castration of the piglets was performed at three days of age using local anaesthesia and analgesia according to a standard protocol with lidocaine 10 mg/ml and epinephrine 5 μg/ml (Xylocain® adrenalin; Aspen Pharma Trading Limited, Dublin, Ireland) and meloxicam 1 mg (Metacam 5 mg/ml; Boehringer Ingelheim Vetmedica GmbH, Ingelheim/Rhein, Germany).

At weaning, all piglets were gathered and driven to the nursery, where the pigs were housed in groups of 150 pigs on deep straw-bedding with access to a concrete floor, outdoor area.

### Study design

In total, eight batches of sows, two per season (summer (June), autumn (October-November), winter (February-mid-March), and spring (mid-March-April)), were included in the study. One batch in each season was assigned to be given an iron supplement and one batch was assigned to not be given an iron supplement. The offspring of the batch assigned to the iron supplement received an intramuscular injection of 200 mg of iron/piglet at three days of age, provided in 1.5 mL of a combined iron and toltrazuril product (Forceris®; Ceva Santé Animale, Libourne, France; 30 mg/mL of toltrazuril and 133.4 mg/mL of iron as gleptoferron). The offspring of the batch assigned to not receive iron supplementation were given an oral treatment with 20 mg toltrazuril/kg bodyweight (Baycoxine® 5%; Bayer Animal Health, Monheim, Germany). Most sows farrowed during the weekends, and thus the first blood sampling for haemoglobin measurement was performed on the subsequent Tuesday when the piglets were herded together for castration and toltrazuril-treatment. On each sampling, the first seven, three-day-old litters selected for castration of male piglets and treatment with toltrazuril were included in the study. Sows of all parities were included in the study, however, sows with health-issues requiring treatment with antibiotics during farrowing were excluded. The piglets were collected from the hut and put in a plastic container. The assisting farmworker collected the piglets from the container randomly, one by one, for sampling. In each litter, seven piglets were selected, except from the first batch where eight pigs were selected. The piglets were individually marked by ear notching. Piglets that were cross-fostered were marked with paint by the farmer when moved and excluded from the study. Male piglets were sampled before castration was performed.

At weaning at 33 days of age, the ear notched pigs were gathered and sampled before being moved into the nursery. Data on sow parity, total number of piglets born, total number of liveborn piglets, number of weaned pigs, and medical treatments of the sows and piglets, were recorded. Data on time of birth (morning, day, evening, night or unknown) were registered. This was defined as: morning, if the farrowing was on-going when the farmer first entered the paddocks in the morning; as day, for farrowings on-going during the mid-day; as evening for farrowings on-going during the evening visit; and night, for farrowings that had not started during the evening visit and were finished at the first morning-visit.

Meteorological data was collected from The Swedish Meteorological and Hydrological institute database [17]. The database consists of temperature values calculated using a gridded analysis model. The longitude and latitude for the position of the herd was entered into the system, and average temperatures for each month during the study period were collected.

### Sampling and haemoglobin analysis

The piglets were restrained and a disposable needle (0.8 × 16 mm, Jorgen Kruuse A/S, Langeskov, Denmark) was used to puncture the auricular vein. The first droplet of blood was removed, and the second droplet was drawn into a microcuvette that was inserted into the analyser and analysed for the haemoglobin concentration.

The haemoglobin concentration was analysed with a HemoCue 201 + Hb photometer (HemoCue AB, Ängelholm, Sweden). The device is developed for the use in human medicine but has been validated for the use in pigs, with an intra-assay coefficient of 1.1 to 2.2% [18]. According to the operating manual, the system is factory-calibrated against the haemiglobincyanide (HiCN) method, the international reference method for the determination of the haemoglobin concentration in blood and hence, no further calibration was needed. The Hb levels were classified as low (Hb < 90 g/L), medium (90 to 110 g/L) or high (H > 110 g/L) [11]. This study did not require official or institutional ethical approval. The animals were handled according to high ethical standards and national legislation.

### Statistical analysis and sample size calculation

The sample size was calculated using Epitools Epidemiological Calculators [19]. To calculate the sample size, a standard deviation of 7.5 and an estimated intra-cluster correlation coefficient (ICC) of 0.5 was applied. Using a 95% confidence level and a precision of 10 g/L, a sample size of seven piglets from seven different litters from each sow group was required.

All data were registered in Microsoft Excel [20] and the statistical analysis were performed using SAS® version 9.4 [21]. Chi-square tests of the difference in distribution of the observations between low, medium, and high Hb levels according to iron supplementation at three days and at 33 days of age, was performed for illustrative purposes. A mixed linear regression model was used to study the Hb levels at three days of age. The model included the fixed effects of season (spring, summer, autumn, or winter), parity (1, 2, 3 or ≥ 4), time of birth (morning, day, evening, night or unknown), gender (male or female), number of live-born piglets and the random effects of batch and of sow nested within batch, to account for the study design. The possibility for a non-linear association between Hb level and number of live-born piglets was assessed by adding the centred and squared value to the model. The effect of iron supplementation on the Hb levels at weaning, i.e. day 33 after birth, was also assessed with a mixed linear regression model. This model added the fixed effects of iron supplementation (yes or no) and Hb levels at day three to the effects already described. Interactions between iron supplementation and the other fixed effects were also introduced in the model, but none were significant and were thus not kept. The goodness-of-fit of the models were checked by graphically assessing the residuals for normal distribution and homoscedasticity and both models showed satisfactory fit. A *P*-value < 0.05 was considered significant.

## Results

In total 56 litters were included in the study, of which 10 litters came from sows included twice in different parities. The number of sows from each parity in the two treatment groups are presented in Table 1.

**Table 1:** Distribution of sows’ parities, grouped according to piglet treatment

	Parity^1^	1	2	3	4	5	6	11	12
Iron supplementation	no^2^	3	4	9	6	1	2	1	2
	yes^2^	8	9	3	5	2	0	1	0
Total number of sows in each parity		11	13	12	11	3	2	2	2

Distribution of sows between different parities, grouped according to if iron supplementation was provided to the piglets at three days after birth or not

^1^ No sows in parity seven to ten were included in the study, and thus these parities are not included in the Table ^2^ number of sows from each parity according to if iron supplementations was provided to the piglets or not (iron yes or iron no)

In total, 399 piglets (204 females, 195 males) were sampled at three days of age, and 378 piglets (199 females, 179 males) were sampled at weaning (33 days of age). Of the 21 missing piglets at weaning, five were recorded as dead, whereas the remaining 16 piglets could not be identified. Out of the 21 piglets, ten had not received iron at three days of age and 11 piglets had received iron at three days of age. The five piglets recorded as dead had all received an iron supplementation at three days of age. Of all piglets sampled, 18 piglets had received an antibiotic treatment during the study period. These were equally distributed between the two treatment groups. Of all sows included in the study, 22 received a treatment with meloxicam 0,4 mg/kg once during or right after farrowing. These were equally distributed between the two treatment groups.

The mean Hb level for all piglets sampled at three days of age was 91 g/L, ranging from 48 g/L to 154 g/L. At weaning, the mean Hb level was 127 g/L, ranging from 76 g/L to 176 g/L. The mean Hb levels at three and 33 days of age, according to whether iron was supplemented or not, are presented in Table 2.

**Table 2:** Haemoglobin concentrations at three and 33 days after birth

	Day 3			Day 33	
	Iron yes	Iron no		Iron yes	Iron no
Mean ± SD	89 ± 16	92 ± 18		122 ± 11	132 ± 16
Median	89	93		123	131
Minimum	52	48		83	76
Maximum	142	154		144	176

Distribution (mean ± standard deviation (SD) and median, minimum, maximum values) of haemoglobin (Hb) concentrations at day three and day 33 (weaning) after birth, according to if iron supplementation was provided to the piglets at three days after birth or not (iron yes or iron no)

The number of pigs with Hb levels classified as low (Hb < 90 g/L), medium (90–110 g/L) or high (Hb > 110 g/L), according to if iron were supplemented or not, are presented in Table 3. No significant differences between the low, medium and high Hb-levels in pigs supplemented with iron, as compared to pigs not supplemented with iron, at three days of age (*P* = 0.10) or at 33 days of age (*P* = 0.21), were indicated.

**Table 3:** Distribution of haemoglobin concentrations at days three and 33 after birth

Hb level	Day 3 (*P* = 0.10)			Day 33 (*P* = 0.21)	
	Iron yes	Iron no		Iron yes	Iron no
Low	102/52%	86/42%		3/2%	1/1%
Medium	74/38%	86/42%		21/11%	14/7%
High	20/10%	31/15%		161/87%	178/92%

Distribution of haemoglobin concentrations at day three and day 33 (weaning) after birth presented as number of pigs / % of pigs. The haemoglobin (Hb) concentrations are classified as low (Hb < 90), medium (90–110) or high (Hb > 110), and according to if iron supplementation was provided or not to the piglets at three days after birth or not (iron yes or iron no). The *P*-values indicate differences in distribution of observations between the low, medium and high values according to iron supplementation

The results from the regression analysis of Hb levels at three days after birth are presented in Table 4. A quadratic term for the number of liveborn piglets was tested but found non-significant and the model had a slightly higher Akaike information criterion (AIC), so the variable was therefore not retained. The total number of piglets, i.e., total liveborn and stillborn, was also evaluated as a potential explanatory variable, but the models had higher AIC’s than the model with only liveborn piglets and was therefore not used. Piglets born in the spring had significantly (*P* < 0.001) lower Hb levels than the piglets born in summer, autumn or winter. Piglets born in autumn had significantly (*P* < 0.001) higher Hb levels than the piglets born in winter, spring or summer.

**Table 4:** Final mixed linear regression model for haemoglobin concentration at day three after birth

Variable	Level	Estimate	Standard error	Overall *P*-value
Intercept		91.8	8.2	
Season	spring	-12.6^a^	5.4	< 0.001
	summer	0.0^b^	Ref	
	autumn	15.9^c^	5.64	
	winter	-2.2^b^	5.5	
Parity	1	2.7	4.0	0.563
	2	0.0	Ref	
	3	5.1	4.0	
	≥ 4	-0.9	3.7	
Time of day, parturition	morning	-10.6	9.0	0.728
	day	-2.7	9.2	
	evening	1.3	3.6	
	night	0.0	Ref	
	not known	3.7	6.0	
Gender	male	0.8	1.2	0.501
	female	0.0	Ref	
Number of live born piglets		-0.3	0.5	0.483

^abc^ Estimates within levels of variables with different superscripts are statistically significant different. Ref indicates the reference level for each variable

Table 5 presents the results of the regression analysis on the Hb levels at day 33 after birth. A significantly lower Hb level could be observed in the group given an iron supplement at three days of age. There were no differences in Hb levels according to season of birth or time of day for parturition, but male piglets had a significantly (*P* = 0.036) lower Hb level than female piglets, but no difference was noted at three days of age (Table 4). The Hb level at three days after birth was significantly positively associated with the Hb level at 33 days after birth.

**Table 5:** Final mixed linear regression model for haemoglobin concentration at weaning

Variable	Level	Estimate	standard error	Overall *P*-value
Intercept		135.1	8.1	
Iron supplement	Yes	-8.6^a^	4.2	0.042
	No	0.0^b^	Ref	
Season	spring	-3.7	6.0	0.098
	summer	0.0^a^	Ref	
	autumn	-2.5	6.1	
	winter	-14.1	6.0	
Parity	1	-2.8	2.7	0.315
	2	0.0	Ref	
	3	0.6	2.7	
	4-	-3.1	2.7	
Time of day, parturition	morning	2.9	5.9	0.090
	day	-3.9	6.2	
	evening	-7.1	2.6	
	night	0.0	Ref	
	not known	-1.4	4.4	
Gender	male	-2.5^a^	1.2	0.036
	female	0.0^b^	Ref	
Number of live born piglets		-0.3	0.3	0.284
Haemoglobin concentration at day 3 after birth		0.1	0.05	0.021

Final mixed linear regression model for haemoglobin concentration at weaning, at day 33 after birth. ^ab^ Estimates within levels of variables with different superscripts are statistically significant different. Ref indicates the reference level for each variable

The marginal means, also known as least-square means, for the Hb level at day 33 after birth, as estimated in the linear mixed regression model, for piglets that received and did not receive iron supplementation was 122.1 ± 3.5 and 130.7 ± 3.3, respectively.

## Discussion

At three days of age, 47% (188 of 399) of the piglets in the present study had Hb levels classified as low (< 90 g/L). The anaemia (Hb levels < 80 g/L) that can be observed during the first days of life is considered to be physiological [2], therefore, the percentage of piglets classified as having low Hb levels were considered to be within the physiological range [2, 3]. However, the physiological anaemia may progress into an IDA if the piglet does not receive a supplement or have access to soil [2, 3, 22]. Therefore, the piglets with low Hb levels at three days of age could be at higher risk of developing an IDA within the first two weeks of life [2, 22]. The piglets had limited access to soil during the first week of life due to the fenders used in front of the hut, and the straw bedding inside the hut. The fenders and the straw bedding might possibly delay the natural supplementation, in the piglets not receiving an iron supplementation at three days of age and increase the risk of development of IDA. However, it is also suggested that molecular regulation of iron absorption in newborn piglets is immature, which could indicate that the access to soil and iron absorption is of less importance during the piglets first week of life [22]. It is not known how fast piglets will regain their iron-deposit and restore their Hb levels when they have direct access to the soil. In a previously performed study, Hb-levels reached 112 g/L at 14 days of age in piglets reared outdoors and not given any iron supplementation [11], indicating that the natural supplementation was sufficient.

At weaning, 90% (339 of 378) of the piglets had Hb levels higher than 110 g/L, classified as high levels, and a mean Hb level of 127 g/L indicating that the majority of the piglets had not developed an IDA [8]. The Hb level at day 33 ranged from 76 g/L to 176 g/L, indicating that there were piglets with IDA at weaning, even though the mean Hb level was classified as high. It is not until the third stage of IDA the effect on blood Hb levels can be measured [1, 7]. Therefore, the initial stages of iron deficiency might be overlooked in the present study [1, 7]. It could be speculated that the iron-levels in the soil has an effect on the Hb levels at weaning. The iron level in the soil has been compared to the blood haemoglobin levels prior to weaning (3–4 weeks of age) in a previous study [9]. No correlation could be observed, although the farm with the lowest mean Hb values in piglets also had the lowest soil iron content [9]. The total iron levels in the soil are generally higher than the available fraction [13], which may be a possible explanation as to why no correlation was found [9]. The Hb level at weaning could also have affected the uptake of iron from the creep feed, but the significance of this iron source is depending on when the piglet starts to eat solid feed, and which amounts of feed that are consumed [3, 23]. To better estimate the iron uptake from the creep feed in the present study, the feed consumption could have been measured.

The Hb levels found in the present study were similar to (127 g/L at 35 days of age) [11] or higher than (111 ± 3.6 g/L at 28 days) [10] the Hb levels found in other studies performed on piglets reared outdoors and not given an iron supplementation. But the results of the present study are also contradiction with previously described results which showed that iron-injected piglets had significantly (*p* < 0.01) higher blood haemoglobin concentration (101 g/L) at weaning (day 34.7 ± 1.8) as compared to the non-injected ones (51 g/L) [14]. The results in the present study are also in contradiction with a study performed in an organic pig herd [23]. In the study, different iron injection regimes were tested and evaluated (one injection on day three; two injections on day three and 14; or three injections on day three, 14 and 21) [23]. The study concluded that one injection was not sufficient and resulted in lower haematocrit and serum iron levels until day 28, as compared to two and three injections. The study also concluded that the haematocrit-levels did not differ between treatment groups at day 14, indicating that the first injection was sufficient for the first 14 days of life [23]. However, it was not stated whether the piglets had access to soil or not, only that the piglets and sows had access to an outdoor area. Therefore, it is not known if differences in the iron-levels in the soil may have caused the contradicting results. There could also be a difference in growth capacity in the piglets included in the studies, resulting in different iron requirements [14].

An interesting finding in the present study was that there was a significant effect of the iron supplementation, leading to a significantly lower Hb level at weaning. A high expression of hepcidin in piglets treated with iron dextran at three days of age, has been shown to be correlated to a suppressed expression of ferroportin in the duodenum [24]. The lower expression of ferroportin might reduce the iron absorption in the intestines [3] and could explain the lower Hb levels at weaning in the piglets given an iron-supplementation at three days of age. However, the difference in Hb levels between iron-supplemented and non-supplemented piglets in the present study is not considered to increase the risk of piglets developing IDA, since 87% and 92% of the piglets in the respective groups reached Hb levels classified as high (> 110 g/L) at weaning.

It could also be argued that the different treatment regimens with injectable toltrazuril given in the iron-supplemented-groups, as compared to an oral toltrazuril given in the non-supplemented groups, could have affected the outcome of the study. However, in a study comparing the two products, the effect of treatment on body weight, faecal consistency and oocyst shedding did not differ significantly [25].

In the regression analysis, a seasonal effect on Hb levels at three days of age was noted. Piglets born in the spring had significantly (*P* < 0.001) lower Hb levels, and piglets born in autumn had significantly (*P* < 0.001) higher Hb levels, than the piglets born during the other seasons. All sows had access to soil from approximately 40 days of pregnancy until farrowing. Significantly higher iron content in the placenta of sows fed a diet high in iron during pregnancy, and numerically but not significantly lower Hb levels in the offspring has been observed [26]. The liver iron content in the offspring was also numerically higher of sows fed a diet with high iron content; 500 μg/g compared to 809 μg/g [26]. Although purely speculative, the amount of soil brought into the hut on the body and the udder of the sow may be higher during warm periods of the year, when the sow spends more time wallowing.

The seasonal effect on Hb levels at day three has not been investigated before. In a previous Swedish study from 2003, a seasonal variation could be demonstrated in piglets at weaning at eight weeks of age [27], indicating a lower Hb level at weaning during winter as compared to summer. Several studies have collected samples from piglets during longer periods of time [11, 14, 15] but no correlation between Hb level and season was investigated. In the present study, no significant association between season and Hb level at weaning was found. However, the results indicates that season may influence Hb levels in piglets reared outdoors, at three days of age, and this needs further investigation. To establish whether a true seasonal effect exists, data from several years need to be included. To rule out specific batch- or farm effects, more herds should also be included.

The regression analysis of Hb levels on day 33 after birth showed that Hb level at three days after birth was significantly positively associated with the Hb level at 33 days after birth. Male piglets had a significantly (*P* < 0.036) lower Hb levels, -2.5 g/L, than female piglets at weaning. It could be speculated that the blood loss due to castration could affect the Hb levels later on, or that differences in growth rate between female and male piglets might have an effect on the iron needs. Further studies are warranted in this respect.

Parity, number of live-born piglets, and time of day for parturition did not have a significant effect on the Hb level at day 33 in the present study. One study focusing on the Hb levels at weaning, demonstrated that piglets from primiparous sows had significantly lower Hb levels as compared to piglets from multiparous sows [12].

We used blood haemoglobin levels to monitor the iron status in the piglets. It has been suggested that using Hb levels as a diagnostic tool may underestimate the iron requirements for young and growing piglets [7]. Three stages of iron deficiency in piglets are described, and it is not until the third stage that the piglet develops an IDA affecting the blood Hb levels [1, 7]. Therefore, the initial stages of iron deficiency might be overlooked [7]. However, in a previous study the results from analysis of additional iron indicators agreed with the Hb measurements and the authors concluded, that the results support the continued use of Hb to monitor iron status in pigs [8]. In addition, it could also be argued that the use of HemoCue is positive from an animal welfare point of view since only a few drops of blood from the ear vein is required to perform the analysis.

## Conclusions

We demonstrated that pigs reared outdoors, will in general obtain sufficient amount of iron through the soil. A seasonal effect on Hb levels at three days of age was observed, but no significant effect on the Hb levels at 33 days of age could be demonstrated. This indicates that there might be a need for different routines regarding iron supplementation in outdoor-reared piglets depending on the climate and season. However, further studies, including several batches per season, more farms and observations collected during several years, are needed to confirm our findings.

## Data Availability

The datasets used and/or analysed during the present study are available from the corresponding author on reasonable request.

## Declarations

### Prior publication

Data have not been published previously.

## Abbreviations

AIC: Akaike Information Criterion
Hb: Haemoglobin
HiCN: Haemiglobincyanide
ICC: Intra–cluster correlation coefficient
IDA: Iron deficiency anaemia
PCV2: Porcine circovirus type 2
SD: Standard deviation

## References

[1] Svoboda M, Drabek J (2005). Iron deficiency in suckling piglets; ethiology, clinical aspects and diagnosis. Folia Vet.

[2] Egeli AK, Framstad T (1998). Evaluation of the efficacy of perorally administered glutamic acid-chelated iron and iron-dextran injected subcutaneously in Duroc and Norwegian landrace piglets. Zentralbl Veterinarmed A.

[3] Szudzik M, Starzynski RR, Jonczy A, Mazgaj R, Lenartowicz M, Lipinski P (2018). Iron supplementation in suckling piglets: an ostensibly easy therapy of neonatal iron deficiency anemia. Pharmaceuticals.

[4] Shields RG, Mahan DC, Graham PL (1983). Changes in swine body composition from birth to 145 kg. J Anim Sci.

[5] Hurley WL, Farmer C (2015). Composition of sow colostrum and milk. The gestating and lactating sow.

[6] Robinson NA, Loynachan AT. Cardiovascular and hematopoietic systems. In: Zimmerman, JJ, Karriker LA, Romirez A, Schwartz KJ, Stevenson GW, Zhang J, editors. Diseases of Swine, 11th Edition. Ames, Iowa: Wiley-Blackwell; 2019;223–33.

[7] Bhattarai S, Nielsen JP (2015). Early indicators of iron deficiency in large piglets at weaning. J Swine Health Prod.

[8] Perri AM, Friendship RM, Harding JCS, O’Sullivan TL (2016). An investigation of iron deficiency and anemia in piglets and the effect of iron status at weaning on post-weaning performance. J Swine Health Prod.

[9] Brown JME, Edwards SA, Smith WJ, Thompson E, Duncan J (1996). Welfare and production implications of teeth clipping and iron injection of piglets in outdoor systems in Scotland. Prev Vet Med.

[10] Kleinbeck AN, McGlone JJ (1999). Intensive indoor versus outdoor swine production systems: genotype and supplemental iron effects on blood haemoglobin and selected immune measures in young pigs. J Anim Sci.

[11] Stern S, Sjölund M, Fellström C, Sterning M, Andersson K. Red blood cell parameters in piglets reared outdoors or indoors. Proceedings of the 16th IPVS Congress, Melbourne, Australia. 2000;192.

[12] Prunier A, Leblanc-Maridor M, Pauwels M, Jaillardon L, Belloc C, Merlot E (2022). Evaluation of the potential benefits of iron supplementation in organic pig farming. Open Res Europe.

[13] Colombo C, Palumbo G, He JZ, Pinton R, Cesco S (2014). Review on iron availability in soil: interaction of Fe minerals, plants, and microbes. J Soils Sediments.

[14] Szabo P, Bilkei G (2002). Iron deficiency in outdoor pig production. J Vet Med Physiol Pathol Clin Med.

[15] Pearson RB. A UK farm study to evaluate productive parameters following routine iron injection of outdoor piglets. Proceedings of the 21st IPVS Congress, Vancouver, Canada. 2012;1036.

[16] Farm. & Animal Health national pig production records database. Farm & Animal Health, Sweden. 2021. https://www.gardochdjurhalsan.se/winpig/medeltal-och-topplistor/medeltal-suggor/. Accessed 20 Oct 2021.

[17] The Swedish meteorological and hydrological database. The Swedish meteorological and hydrological institute. 2023. https://www.smhi.se/data/ladda-ner-data/griddade-nederbord-och-temperaturdata-pthbv. Accessed 6 Jul 2023.

[18] Kutter APN, Mauch JY, Riond B, Martin-Jurado O, Spielmann N, Weiss M (2012). Evaluation of two devices for point-of-care testing of haemoglobin in neonatal pigs. Lab Anim.

[19] Sergeant ESG. Epitools epidemiological calculators. Ausvet. 2018. Available from: http://epitools.ausvet.com.au

[20] Microsoft Corporation. Microsoft Excel 2018. Available from: https://office.microsoft.com/excel

[21] SAS® version 9.4 (SAS Institute Inc. Cary, USA).

[22] Lipiński P, Starzyński RR, Canonne-Hergaux F, Tudek B, Oliński R, Kowalczyk P (2010). Benefits and risks of iron supplementation in anemic neonatal pigs. Am J Pathol.

[23] Heidbüchel K, Raabe J, Baldinger L, Hagmüller W, Bussemas R (2019). One iron injection is not enough - iron status and growth of suckling piglets on an organic farm. Animals.

[24] Pu Y, Guo B, Liu D, Xiong H, Wang Y, Du H (2015). Iron supplementation attenuates the inflammatory status of anemic piglets by regulating hepcidin. Biol Trace Elem Res.

[25] Joachim A, Shrestha A, Freudenschuss B, Palmieri N, Hinney B, Karembe H (2018). Comparison of an injectable toltrazuril-gleptoferron (Forceris®) and an oral toltrazuril (Baycox®) + injectable iron dextran for the control of experimentally induced piglet cystoisosporosis. Parasites Vectors.

[26] Buffler M, Becker C, Windisch WM (2017). Effects of different iron supply to pregnant sows (*Sus scrofa Domestica* L.) on reproductive performance as well as iron status of new-born piglets. Arch Anim Nutr.

[27] Beskow P, Norqvist M, Lundeheim N, Wallgren P (2003). Utomhusproduktion Av Grisar i Norrland. Svensk Veterinärtidning.

